# Molecular profile of cochlear immunity in the resident cells of the organ of Corti

**DOI:** 10.1186/s12974-014-0173-8

**Published:** 2014-10-14

**Authors:** Qunfeng Cai, R Robert Vethanayagam, Shuzhi Yang, Jonathan Bard, Jennifer Jamison, Daniel Cartwright, Youyi Dong, Bo Hua Hu

**Affiliations:** Center for Hearing and Deafness, State University of New York at Buffalo, 137 Cary Hall, 3435 Main Street, Buffalo, NY 14214 USA; Department of Otolaryngology, The first affiliated Hospital to Chinese PLA General Hospital, 51, Fucheng Road, Haidian District, Beijing, 100048 China; Next-Generation Sequencing and Expression Analysis Core, New York State Center of Excellence in Bioinformatics and Life Sciences, State University of New York at Buffalo, 701 Ellicott Street, Buffalo, NY 14260 USA

**Keywords:** Immunity, The organ of Corti, Noise, Sensory cells, Inflammation, Supporting cells

## Abstract

**Background:**

The cochlea is the sensory organ of hearing. In the cochlea, the organ of Corti houses sensory cells that are susceptible to pathological insults. While the organ of Corti lacks immune cells, it does have the capacity for immune activity. We hypothesized that resident cells in the organ of Corti were responsible for the stress-induced immune response of the organ of Corti. This study profiled the molecular composition of the immune system in the organ of Corti and examined the immune response of non-immune epithelial cells to acoustic overstimulation.

**Methods:**

Using high-throughput RNA-sequencing and qRT-PCR arrays, we identified immune- and inflammation-related genes in both the cochlear sensory epithelium and the organ of Corti. Using bioinformatics analyses, we cataloged the immune genes expressed. We then examined the response of these genes to acoustic overstimulation and determined how changes in immune gene expression were related to sensory cell damage.

**Results:**

The RNA-sequencing analysis reveals robust expression of immune-related genes in the cochlear sensory epithelium. The qRT-PCR array analysis confirms that many of these genes are constitutively expressed in the resident cells of the organ of Corti. Bioinformatics analyses reveal that the genes expressed are linked to the Toll-like receptor signaling pathway. We demonstrate that expression of Toll-like receptor signaling genes is predominantly from the supporting cells in the organ of Corti cells. Importantly, our data demonstrate that these Toll-like receptor pathway genes are able to respond to acoustic trauma and that their expression changes are associated with sensory cell damage.

**Conclusion:**

The cochlear resident cells in the organ of Corti have immune capacity and participate in the cochlear immune response to acoustic overstimulation.

## Background

The organ of Corti in the auditory sensory epithelium of the cochlea contains sensory hair cells and their supporting cells. The sensory cells are mechanoreceptors for hearing. Compared with other cell populations in the cochlea, these sensory cells are susceptible to pathological insults [1-3]. When damage to these cells exceeds their capacity to repair, cell death ensues. Morphological observations have revealed the rapid degradation and removal of dead sensory cells [1]. This rapid clearance of damaged cells from the cochlea is essential to protect the surviving cells from toxic molecules released from damaged or dead cells.

In the cochlea, the molecular processes for monitoring sensory cell damage and removing dead cells are not clear. Emerging evidence has implicated the immune response in many inner ear disorders [4-7]. The immune response is an essential biological process for tissue defense and cell survival. This defense mechanism protects cells against not only bacterial and viral invasion but also against endogenous host-derived molecules from damaged cells and tissues.

The immune response is mediated by immune cells. However, several pieces of evidence suggest that the stress-induced immune response of the organ of Corti, the most vulnerable site in the cochlea, is not mediated by immune cells. First, migration of immune cells into the cochlea occurs after acoustic trauma [8]. These cells exhibit markers typical for immune cells, including CD45 [9,10], F4/80 [9] and IBA1 [10,11]. However, these migrating immune cells primarily accumulate in tissue that is not part of the organ of Corti, including the spiral ligament and the scala tympani [9,12-15]. Second, resident immune cells in the cochlea participate in cochlear responses to stress. However, these cells reside in the non-organ of Corti tissues, including the spiral ligament and the spiral ganglion [16]. Despite the lack of immune cells, a handful of immune molecules have been identified in the organ of Corti [17-20], suggesting that the resident cells in the organ of Corti have immune capacity. To date, the molecular composition of the immune system in the organ of Corti remains largely unknown. A better understanding of the immune capacity of these non-immune epithelial cells is necessary to elucidate cochlear responses to stress.

This study sought to define the molecular composition of the immune system in cells of the organ of Corti and to determine how those cells respond to stress. We profiled the expression pattern of a large set of immune and inflammatory genes from tissue that was isolated from the organ of Corti using a micro-dissection technique recently developed in our laboratory [21]. We confirmed the expression results with RNA-sequencing (RNA-seq) analysis of data from the cochlear sensory epithelium. Bioinformatics analyses linked multiple immune-related signaling pathways, including the Toll-like receptor signaling pathway, to the expressed genes. We further demonstrated that supporting cells in the organ of Corti are the major source of the expression of Toll-like receptor signaling-related genes. Importantly, these genes respond to cochlear acoustic stress, and changes in their expression are associated with sensory cell damage. Collectively, these findings indicate that the resident cells in the organ of Corti have immune capacity and are able to participate in the cochlear immune responses to acoustic stress.

## Materials and methods

### Animals

C57BL/6 J, CBA/CaJ and B6.B10ScN-Tlr4^lps-del^/JthJ mice (4 to 8 weeks, male and female, The Jackson Laboratory, Bar Harbor, ME, USA) were used. CBA mice were used in the RNA-seq analysis because of their late-onset of cochlear degeneration with age. C57BL/6 J mice were used in remaining experiments because this strain is a commonly used control for genetically modified mouse strains including the B6.B10ScN-Tlr4^lps-del^/JthJ mouse used in the current study. All animals received a baseline hearing evaluation. Only mice that exhibited normal hearing sensitivity were included in the study. To facilitate data presentation, the number of animals (cochleae) used for each experiment is described in the Results section.

All procedures involving the use and care of the animals were approved by the Institutional Animal Care and Use Committee of the State University of New York at Buffalo.

Previous studies have demonstrated that C57BL/6 J mice display early onset of age-related hearing loss [22-24]. To prevent the influence of this confronting factor on noise-induced cochlear damage, we used mice with the ages ranging from four to eight weeks. Mice in this age range display relatively normal hearing sensitivity [24].

### Experimental procedures

The animals received a baseline hearing evaluation and then were assigned to an experimental group. For the experiments profiling immune-gene expression, the samples were collected from normal cochleae. For the experiments to determine noise-induced changes in immune-related gene expression, the animals were exposed to an intense noise and the cochleae were collected 1 day or 4 days after the noise exposure. To determine the permanent noise damage to the cochleae, the auditory brainstem responses (ABR) were measured and the cochleae of the mice were collected for pathological assessment at 14 days after the noise exposure.

### Tissue collection

Based on the aims of each experiment, defined cochlear samples were collected. For the RNA-seq analysis, the sensory epithelium was collected. For the qRT-PCR array and the individual qRT-PCR analysis, the organ of Corti was collected. A detailed description of cell composition of these samples is provided in the following sections. For the Western blotting analysis of cochlear tissues, the total intra-cochlear tissues from four cochleae were pooled to generate one sample.

For all of the sample collections, animals were decapitated under deep anesthesia with CO_2_. Within 2 minutes after the beginning of the CO_2_ inhalation, the cochleae were quickly removed from the skull and processed using one of the following methods for the sample collection.

### Collection of the sensory epithelium for RNA-seq analyses

**Figure 1:**
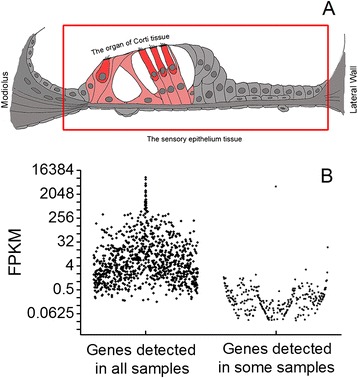
**The regions of sample collection and expression levels of immune genes. (A)** Schematic drawing of a cross-section of the cochlear sensory epithelium. The red rectangle marks the cell populations of the sensory epithelium that were collected for the RNA-seq analysis. The cells in red or pink comprise the organ of Corti, which consists of sensory hair cells (red) and supporting cells (pink). This tissue was collected for qRT-PCR array and individual qRT-PCR analysis of immune gene expression. **(B)** Expression pattern of immune/inflammatory genes in the cochlear sensory epithelium. Comparison of the expression levels of the immune genes that were detected in all examined samples with those that were detected in some samples. One dot represents the expression of one gene. The genes detected in all samples display a higher average expression level than those that were detected in some samples (Mann-Whitney Rank Sum Test, *P* <0.001).

The sensory epithelium contains all of the cells on the basal membrane between the lateral wall and the modiolus (Figure 1A). This tissue was collected from the cochlear region approximately 50 to 80% from the apex. This region corresponds to the upper first cochlear turn. We selected this region for the expression analysis because this region displayed consistent sensory cell damage. Moreover, this region has been used in our previous investigations for analyses of molecular changes after acoustic trauma [21,25]. Analysis of the same region enabled us to compare the data collected from different studies. We intentionally avoided using the tissues from the basal extreme (>80% from the apex) to prevent the potential influence of age-related sensory cell degeneration, which starts at the basal extreme.

After the animals were sacrificed, the cochlea was quickly removed and placed in an RNA-stabilizing reagent (RNAlater; Qiagen, Valencia, CA, USA). The bony shell of the cochlea was opened and the apical turn was removed to expose the basal turn of the cochlea. The lateral wall of the cochlea was removed using a fine needle. Then, the sensory epithelium was gently separated from the modiolus of the cochlea. The isolated tissue was transferred to an RNase-free PCR tube and stored at −80°C before the subsequent analysis of gene expression.

### Collection of the organ of Corti tissues for transcriptional analyses

The organ of Corti contains sensory cells (inner hair cells and outer hair cells) and supporting cells (Deiters cells, pillar cells, Hensen cells, inner phalangeal cells and inner border cells) (Figure 1A). The tissue was collected from the basal turn of the cochlea. Each sample contained the tissue collected from one cochlea.

The method of the tissue collection has been described in our recent publication [21]. Briefly, after the animals were sacrificed, the cochlea was quickly removed and placed in ice-cold Dulbecco’s PBS (DPBS, GIBCO, Life Technologies, Grand Island, NY, USA). The bony shell facing the middle ear cavity was quickly removed and the cochlea was placed in RNAlater solution. Using a custom-made micro-knife, we gently scraped the reticular laminar at the junction between the Deiters cells and the Hensen cells and pushed the tissue away from the basilar membrane. The isolated tissue was transferred to a small dish containing fresh RNAlater solution to wash out tissue debris from the surface of the sample. Then, the tissue was transferred to an RNase-free PCR tube. The sample was stored at −80°C before the subsequent analysis of gene expression.

### Collection of cochlear tissue and other mouse tissues for Western blotting

Cochlear tissue for Western blotting contained all intra-cochlear tissues including the modiolus, the sensory epithelium and the lateral wall. The tissues were pooled from four cochleae to generate a sufficient sample size for Western blotting analysis. After the animals were sacrificed, the cochlea was quickly removed from the skull and placed in ice-cold 10 mM phosphate-buffered saline (PBS, Sigma-Aldrich, St. Louis, MO, USA). The bony shell of the cochlea was removed. All intra-cochlear tissues were collected and transferred to a PCR tube. In addition, we collected mouse brain, kidneys, spleen and intestine. The samples were stored at −80°C before the subsequent analysis.

### Collection of cochlear tissue for immunohistology

After the animals were sacrificed, the cochlea was collected and placed in ice-cold 10 mM PBS. The round window and the oval window of the cochlea were opened with a fine needle. Through the round window, 10% buffered formalin was gently perfused into the cochlea. The cochlea was placed in the same fixative for two hours and then rinsed with PBS. The cochlea was dissected in PBS to collect the sensory epithelium under a dissection microscope.

### RNA sequencing

RNA-seq analyses were performed to define the expression profile of immune/information related genes in the cochlear sensory epithelium. The total RNA was extracted from the sensory epithelium using the Qiagen RNeasy Micro Kit (Qiagen GmbH, Hilden, Germany) as per the manufacturer’s protocol. The quality and quantity of total RNA was evaluated using the Agilent Bioanalyzer 2100 (Agilent Technologies, Santa Clara, CA, USA). The RNA Integrity Number (RIN) and the total RNA concentration were determined for all eight samples.

The synthesis of cDNA from the total RNA for each sample was performed with the Clontech SMARTerTM Ultra Low RNA Kit (Clontech Laboratories Inc., Mountain View, CA, USA). From each cDNA sample, a sequencing library was prepared using the Illumina Paired End Sample Prep Kit (Illumina Inc., San Diego, CA, USA) according to the Illumina Ultra Low Input mRNA-Seq Protocol. The average insert size of the libraries was 124 bp. Each cDNA library was sequenced in a 50-cycle single read flow cell lane on an Illumina HiSeq 2000. Four biological repeats were performed for each sample generated from a single cochlear sensory epithelium.

For the RNA-seq data analysis, the sequence results were aligned to the mouse reference genome sequence (USCS Genome Browser, MM10) using TopHat version 1.3.2 [26] and Bowtie [27]. The resulting alignments were further assembled and annotated using Cufflinks software [28]. The abundance of the gene expression was normalized to the fragments per kilobase of exon model per million mapped reads (FPKM) [29]. To compare the average expression levels of the genes that were detected in all examined samples and the genes that were detected in some of the examined samples, a Mann-Whitney Rank Sum Test was performed.

### qRT-PCR array

qRT-PCR array analysis was performed to profile the expression pattern of immune/informatory genes in the normal organ of Corti and to determine the changes in gene expression after acoustic trauma. For the gene profiling of normal tissue, we used two types of array plates: mouse innate/adaptive gene plates and mouse inflammatory cytokine/receptor gene plates (PAMM-052ZD and PAMM-011ZA, Qiagen, Valencia, CA, USA), each containing 84 target genes and 5 reference genes. For the analysis of noise-induced expression changes, the mouse innate/adaptive gene plates were used. Three biological repeats were performed for each experimental condition.

The protocol to isolate the total RNA, synthesize and pre-amplify the cDNA and run the qRT-PCR reactions was performed as previously described [21]. Total RNA was isolated from each sample using the RNeasy Micro Kit (Qiagen, Valencia, CA, USA) as per the manufacturer’s instructions. The isolated total RNA was used to generate cDNA, and the cDNA was pre-amplified using the RT^2^ Nano PreAMP cDNA Synthesis Kit (Qiagen, Valencia, CA, USA). The synthesized cDNA was mixed with RT^2^ Real-Time PCR SYBR Green/Fluorescein Master Mix (Qiagen, Valencia, CA, USA), transferred to a 96-well plate and used to perform qRT-PCR on a Bio-Rad CFX Real-Time PCR System. The quality control for the mRNA quantification was performed using three integrated control assays in the PCR array: a reverse transcription control, a positive PCR control, and a genomic DNA control. All PCR runs passed the control tests.

For analysis of the expression pattern of immune genes in the normal organ of Corti, the Ct (the cycle threshold) values of the target genes were normalized to the average expression level of five reference genes (*Actb*, *B2m*, *Gapdh*, *Gusb* and *Hsp90ab1*) to generate the ΔCt values. For analysis of noise-induced expression changes, a relative quantification method [30] was used to evaluate the change in expression levels of mRNA following exposure. The expression level of a given gene was first normalized to the average level of the reference genes to generate the ΔCt of each target gene. Then, the ΔΔCt was calculated with the formula:

$$ \Delta \Delta \mathrm{C}\mathrm{t}=\varDelta \mathrm{C}\mathrm{t}\ \left(\mathrm{noise}\ \mathrm{group}\right)-\varDelta \mathrm{C}\mathrm{t}\left(\mathrm{control}\ \mathrm{group}\right) $$

One-way analysis of variance (ANOVA) was performed to compare the expression levels of the samples collected from the control, 1 day post-noise and 4 days post-noise groups. The Tukey *post hoc* test was used to evaluate the differences between pairs (pre- versus 1 day and pre- versus 4 day).

### Individual mRNA expression analysis

Taqman individual qRT-PCRs were performed to confirm the results of the PCR array analysis of noise-induced changes in *Tlr3* and *Tlr4* expression in the organ of Corti. Again, the tissue from one cochlea was used to generate one sample. The total RNAs were extracted from the organ of Corti using the method described above for the qRT-PCR array analysis. The isolated total RNAs were reverse transcribed using a high capacity cDNA reverse transcription kit (product catalog number: 4374966, Applied Biosystems, Foster City, CA, USA). qRT-PCR was performed on a MyIQ two color Real-Time PCR detection system (Bio-Rad, Hercules, CA, USA). Four biological repeats were performed for each experimental condition (noise and control).

For the analysis of the changes in expression of *Tlr3 and Tlr4*, we first examined the expression levels of three reference genes (*Hprt, Hsp90ab, Rpl13a*). Then, the expression levels of *Tlr3* and *Tlr4* were normalized to the average level of these reference genes. Student’s *t*-test was used to compare the expression levels between the control and the 1 day post-noise groups.

### Immunohistology

Immunohistochemistry was performed to examine the cell-specific expression of immune-related proteins (TLR3, TLR4, IRF7 and STAT1), to illustrate the immune cell distribution (CD45 protein) and to visualize the cell structure (β-tubulin) or outer hair cells (prestin protein) in the organ of Corti or the sensory epithelium. The cochleae were fixed using 10% buffered formalin. After dissection in PBS, the sensory epithelium was collected. The tissues were then permeabilized with 0.2% Triton X-100 in PBS for 30 to 60 minutes, blocked with a blocking buffer (Table 1) for 1 hour and then incubated overnight at 4°C with a primary antibody or 2 primary antibodies (for double-labeling of 2 proteins) at a concentration recommended by the manufacturer (Table 1). The tissues were then rinsed with PBS (3×) and incubated with a secondary antibody for 2 hours (Table 1). Unless a double-staining was performed for 2 proteins, all immunolabeled tissues were counterstained with propidium iodide (5 μg/ml in PBS) for 10 minutes. The tissues were mounted on slides with an antifade medium (Prolong® Gold antifade reagent, Invitrogen, Carlsbad, CA, USA).

**Table 1:** **Antibodies used for immunohistology**

	**Protein**	**Primary antibody**	**Secondary antibody**
1	Tlr3	Anti-TLR3 antibody (ab62566 Abcam)	Alexa Fluor 488 donkey anti rabbi
2	Tlr4	Anti-TLR4 antibody (ab13556 Abcam)	Alexa Fluor 488 donkey anti rabbit
3	Irf7	Anti-IRF7 antibody (ab109255 Abcam)	Alexa Fluor 488 donkey anti rabbit
4	Stat1	Anti-STAT1 antibody (ab2415 Abcam)	Alexa Fluor 488 donkey anti rabbit
5	CD45	CD45 Antibody (AF114 R&D Systems, Inc.)	Alexa Fluor 488 or 568 donkey anti goat
6	β-tubulin	β Tubulin (sc-9935 Santa Cruz Biotechnology, Inc.)	Alexa Fluor 568 donkey anti goat
7	prestin	Prestin Antibody (Sc-22692 Santa Cruz Biotechnology, Inc.)	Alexa Fluor 568 donkey anti goat

To quantify the changes in Tlr4 expression after acoustic trauma, the tissues were photographed using confocal microscopy (Zeiss, LSM 510, Thornwood, NY, USA). The staining intensity of Tlr4 was measured with image processing software (IMAGE-Pro Plus, Media Cybernetics Co., Rockville, MD, USA). Specifically, the gray levels of the pixels within the cytoplasm of the Deiters cells beneath the damaged hair cells and the neighboring Deiters cells beneath the surviving hair cells were quantified. Three to five cells for each type of Deiters cells were measured in each cochlea. Then, the gray levels for individual cells were averaged to generate a mean value for each condition (beneath the damaged outer hair cells versus beneath the surviving outer hair cells). The differences in the gray levels were statistically analyzed using Student’s *t*-test.

All of the immunostaining analyses had proper controls. For the assessment of the changes in the expression patterns of immune proteins following acoustic trauma, the cochleae from the control animals that did not undergo noise exposure were used as the normal control. For the immune-protein staining, immune cells in the cochlea were used as positive controls. To verify the specificity of the antibodies, Western blotting was performed to determine the molecular weights of the proteins targeted by the antibodies. Several pieces of the organs of Corti from the normal cochleae were stained with only the secondary antibodies to assess nonspecific staining.

### Confocal microscopy

The immunolabeled tissues were first inspected with a microscope equipped with epifluorescence illumination to identify sensory cell lesions. Sites of interest were further examined with confocal microscopy (LSM510 multichannel laser scanning confocal imaging system, Zeiss, Thornwood, NY, USA) using a method that has been reported previously [31,32].

### Assessment of sensory cell damage

Hair cell morphology was examined to determine noise-induced sensory cell damage in the basal turn of the cochlea. To reduce dissection-induced tissue damage, we used a method of *in situ* observation of the cochlea developed recently in our laboratory. The cochlea was fixed with 10% buffered formalin. The apical section of the cochlea was removed to expose the basal turn of the cochlea. Using a fine needle, the lateral wall tissue was removed. To visualize cell structures, either Alexa Fluor 488 phalloidin staining or prestin staining was performed. For the phalloidin staining, the cochlea was incubated with the staining solution containing Alexa Fluor 488 phalloidin (Applied Biosystems, Carlsbad, CA, USA; 1:250), 0.25% Triton X-100 and 1% BSA in PBS at room temperature for 30 min. For prestin staining, the cochlea was immunolabeled using a method that has been described in the above Immunohistology section.

After the staining, the cochlea was rinsed in PBS and placed in a culture dish containing distilled water for microscopic observation. Sequential images of 3 to 15 layers covering the entire depth of the sensory epithelium for each section were taken using a digital camera (SPOT RT, Color Diagnostic Instruments, Inc., Sterling Heights, MI, USA). Using Adobe Photoshop CS6, individual images were aligned and the images of different layers were blended to generate a merged view of the tissue. The images were visually inspected. Missing phalloidin staining in the cuticular plates or missing prestin staining in outer hair cells were considered as missing cells. The number of missing outer hair cells was quantified by a single observer and this observer was not blinded to the experimental conditions. The data were presented in a cochleogram.

### Bioinformatics pathway analysis

The bioinformatic analysis of immune genes that were expressed in the organ of Corti was performed to determine their relevant signaling pathways. KEGG-pathway and Panther-pathway analyses were performed through the DAVID database (the database of annotation, visualization and integrated discovery) [33]. Based on the *P*-values of each pathway, we selected the most relevant pathways for further experimental confirmation.

To determine which mouse genes are immune and inflammation-related genes, we searched the MGI database (Mouse Genome Informatics). Two general ontology terms, ‘immune system process’ and ‘inflammatory’, were used for this data inquiry.

### Western blotting

Western blotting was used to confirm the specificity of the antibodies used in the current investigation. Whole cell lysates from the cochlear, brain, spleen, kidney and intestinal tissues were prepared using a radio-immunoprecipitation assay (RIPA) lysis buffer system (Santa Cruz Biotechnology, Dallas, TX, USA; catalog number: sc-24948) and the lysates were centrifuged at 12,000 rpm at 4°C for 15 minutes. The protein concentrations of the supernatants were determined by the Bio-Rad quick start Bradford protein assay (Bio-Rad, Hercules, CA, USA). Total proteins from the supernatants were separated on a 12% acrylamide gel, and the proteins were electrophoretically transferred onto polyvinylidene difluoride (PVDF) membranes. After blocking with Tris-buffered saline with 0.5% non-fat dry milk, the blots were incubated with a primary antibody overnight at 4°C. The following primary antibodies were used: TLR3 (Abcam, catalog number: ab62566; Brain-auditory region was used as positive control), TLR4 (Abcam, Cambridge, MA, USA; catalog number: ab13556; mouse spleen, intestine and kidney were used as positive controls), IRF7 (Abcam, catalog number: ab109255; the mouse cochlea was used) and STAT1 (Abcam, catalog number: ab2415; the mouse cochlea was used). The membranes were then incubated with a horseradish peroxidase-conjugated secondary antibody, the blots were rinsed, and the protein bands were visualized using an enhanced chemiluminescence detection system (Thermo scientific, Rockford, IL, USA).

### Noise exposure

A continuous noise (1 to 7 kHz) at 120 dB (sound pressure level, re 20 μPa) for 1 hour was used to traumatize the cochlea. This level of noise exposure was selected because it is able to cause permanent hearing loss and sensory cell death [21], allowing us to determine the cochlear immune response to sensory cell damage. The noise signal was generated using a Real-Time signal processor (RP2.1, Tucker Davis Technologies, TDT, Alachua, FL, USA). The signal was routed through an attenuator (PA5 TDT, Alachua, FL, USA) and a power amplifier (Crown XLS 202, Harman International Company, Elkhart, IN, USA) to a loudspeaker (NSD2005-8, Eminence, Eminence, KY, USA) positioned 30 cm above the animal’s head. The noise level at the position of the animal’s head in the sound field was calibrated using a sound level meter (LD-PCB, model 800 B, APCB Piezotronics Div., Larson Davis, Depew, NY, USA), a preamplifier (LD-PCB, model 825, Larson Davis, Depew, NY, USA), and a condenser microphone (Larson and Davis, LDL 2559, Depew, NY, USA). The mice were individually exposed to the noise in a holding cage.

### Auditory brainstem responses (ABR)

ABR measurements were conducted to assess the auditory function of the mice. The ABRs were recorded as previously described [25]. Briefly, an animal was anesthetized with an intraperitoneal injection of a mixture of ketamine (87 mg/kg) and xylazine (3 mg/kg). The body temperature was maintained at 37.5°C with a warming blanket (Homeothermic Blanket Control Unit, Harvard Apparatus, Holliston, MA, USA). Stainless-steel needle electrodes were placed subdermally over the vertex (noninverting input) and posterior to the stimulated and nonstimulated ears (inverting input and ground) of the animal. The ABRs were elicited with tone bursts at 4, 8, 16, and 32 kHz (0.5 ms rise/fall Blackman ramp, 1 ms duration, alternating phase) at the rate of 21/s, which were generated digitally (SigGen, Alachua, FL, USA) using a D/A converter (RP2.1; TDT; 100 kHz sampling rate) and fed to a programmable attenuator (PA5; TDT), an amplifier (SA1; TDT), and a closed-field loudspeaker (CF1; TDT). The electrode outputs were delivered to a pre-amplifier/base station (RA4LI and RA4PA/RA16B; TDT). The responses were filtered (100 to 3,000 Hz), amplified and averaged using TDT hardware and software. These responses were then stored and displayed on a computer. The ABR threshold was defined as the lowest intensity that reliably elicited a detectable response. The ABR measurements were performed by a single observer and the observer was not blinded to the experimental conditions.

The average ABR thresholds obtained pre- and post-noise exposure or between the wild-type and *Tlr4* knockout mice were compared using two-way ANOVA with two factors of either time × frequency or species × frequency. If significant main effects were identified, the Tukey *post hoc* test was used to evaluate the interaction between the main factors.

### Genotyping

Mouse genotyping was performed to verify the genotype of the B6.B10ScN-Tlr4^lps-del^/JthJ mice. Genomic DNA was extracted from the tail of the knockout mice and wild-type mice (C57BL/6 J) using a PCR lysis kit (the Direct PCR lysis kit, Viagen biotech, Los Angeles, CA, USA) according to the manufacturer’s instructions. Genotyping was performed via standard PCR using genomic DNA as the template. The PCR primers used were: 5′- GCA AGT TTC TAT ATG CAT TCT C- 3′ (Mutant forward); 5′- CCT CCA TTT CCA ATA GGT AG -3′ (Mutant reverse); 5’- ATA TGC ATG ATC AAC ACC ACA G -3’ (Wild-type forward); 5’-TTT CCA TTG CTG CCC TAT AG-3’ (Wild-type reverse). *Taq* DNA polymerase (Invitrogen, Carlsbad, CA, USA) was used in the PCR reaction. The cycling conditions consisted of a 94°C initial denaturation step for 3 minutes; 34 cycles of 94°C for 30 s, 55°C for 1 minute and 72°C for 1 minute; and a final extension step of 72°C for 2 minutes. The PCR products were subjected to 1.5% agarose gel electrophoresis and were visualized by ethidium bromide staining.

### Data analyses

All functional and molecular comparisons were statistically analyzed using SigmaStat (Version 3.5) (San Jose, CA, USA) or GraphPad Prism (Version 5) (La Jolla, CA, USA). An α-level of 0.05 was selected for significance for all statistical tests.

## Results

### RNA-seq reveals the constitutive expression of immune response-related genes in the cochlear sensory epithelium

The cochlea consists of three partitions, the sensory epithelium, the lateral wall and the modiolus. The sensory epithelium contains the sensory cells (Figure 1A), which are the major target of pathological insults. To define the expression pattern of immune and inflammatory genes in the sensory epithelium, we used next-generation RNA-seq to obtain a digital inventory of the mRNA transcriptome (four biological repeats from four individual subjects).

To determine which genes have been implicated in immune and inflammatory processes in the mouse, we searched the Mouse Genome Informatics database for annotated genes linked to the biological processes of immunity and inflammation. Two ontology terms, ‘immune system process’ and ‘inflammatory’ were used for this inquiry because they are the most general terms in the biological process ontology for the immune and inflammatory processes. We found 1,633 genes that linked to the immune system and inflammatory processes in the mouse. Among these, 1,592 genes were annotated in the Genome Reference Consortium Mouse Build 38(GRCm38)/mm10 assembly that was used for mapping the annotated genes in this investigation.

We then determined which of the annotated immune/inflammatory genes were expressed in the cochlear sensory epithelium. To reduce the false-positive identification of gene transcripts, we set the cut-off value to remove low abundance genes (fragments per kilobase of exon model per million mapped reads (FPKM) >0.1). FPKM values below 0.1 represent low to undetectable gene transcript levels [34,35]. There were 967 genes detected in all four samples and 341 genes that were not detected in any sample. The remaining 284 genes were detected in some of the samples. The average expression level of these inconsistently detected genes was lower than that of the genes that were detected in all samples (Figure 1B, Mann-Whitney Rank Sum Test, *P* <0.001). This is agreement with a previous observation that the expression of low abundance genes is less consistent [29]. Because of this inconsistency, we were unable to define their expression pattern. Therefore, we focused subsequent analyses on genes that were expressed in all samples.

These expressed genes exhibited diverse expression levels. A few genes exhibited exceptionally high or low expression levels. Table 2 lists the 60 genes with the highest expression and the 30 genes with the lowest expression. The RNA-seq analyses revealed robust expression of immune and inflammatory genes in the mouse sensory epithelium.

**Table 2:** **Expression levels of highly and weakly expressed immune/inflammation-related genes in the normal cochlear sensory epithelium**

**Rank**	**Symbol**	**Expression levels (FPKM)**		**Rank**	**Symbol**	**Expression level (FPKM)**	
		**Mean**	**SD**			**Mean**	**SD**
**Highly-expressed genes**							
1	Spp1	8,974.98	2,782.16	31	Hbb-b2	568.64	366.51
2	Rps14	7,445.93	4,423.91	32	Gapdh	541.79	128.01
3	Gpx2	5,096.75	788.79	33	Tmem176a	506.73	52.39
4	Apoe	4,413.41	1,207.30	34	Vimp	455.65	61.00
5	Trf	4,209.45	2,351.89	35	Bcap31	455.02	43.53
6	Hba-a2	2,849.79	1,583.28	36	Atpif1	436.87	35.55
7	Gpx4	2,757.61	865.85	37	Nfkbia	377.97	91.52
8	Rps19	2,593.07	868.93	38	Hmgb1	376.21	22.02
9	Lgals1	2,375.43	434.34	39	Pla2g7	362.19	109.04
10	Rps6	1,867.83	447.50	40	Serpinf1	346.23	188.45
11	Rpl13a	1,751.85	658.77	41	Phpt1	340.86	71.68
12	Prdx1	1,711.38	428.22	42	Gnas	339.87	88.70
13	Nme2	1,278.45	281.30	43	Cyba	335.33	47.53
14	Selk	1,253.00	297.47	44	Psme2	329.11	94.80
15	Ifitm2	1,119.77	252.30	45	Fkbp1a	327.71	33.60
16	Car2	1,063.46	490.25	46	Tsc22d3	319.84	88.51
17	Rps24	1,038.78	447.60	47	Calr	307.89	79.13
18	Cd81	1,032.72	196.45	48	Nme1	280.60	45.34
19	B2m	1,003.31	23.57	49	Fcgrt	275.06	19.30
20	Prdx2	983.57	113.75	50	Rgcc	265.59	43.20
21	Tmem176b	914.41	114.35	51	Hprt	250.51	56.23
22	Park7	900.11	33.82	52	Prelid1	225.27	44.14
23	Ifitm3	871.64	30.05	53	Bnip3	225.24	41.48
24	Crip2	828.16	122.21	54	Psmb4	224.41	32.60
25	Sod1	789.94	43.52	55	Psma1	216.16	29.35
26	Fam213a	760.94	148.39	56	S100a8	204.00	138.29
27	Mif	749.95	123.46	57	Bnip3l	200.30	28.93
28	Id2	645.03	97.69	58	Wdr61	199.40	33.21
29	Gpx1	617.58	229.02	59	Hsp90aa1	198.63	48.34
30	Ndfip1	611.79	51.31	60	Gstp1	198.18	15.56
**Weakly-expressed genes**							
1	Ptprj	0.45	0.12	16	Il2ra	0.31	0.13
2	Sh2b2	0.44	0.28	17	Nbeal2	0.30	0.16
3	Adar	0.44	0.15	18	Cnr2	0.28	0.06
4	Camk1d	0.43	0.21	19	Cxcr4	0.27	0.12
5	Tcf7	0.42	0.38	20	Tbkbp1	0.27	0.16
6	Lcp2	0.42	0.25	21	C4b	0.27	0.25
7	Rnasel	0.40	0.29	22	Myo1f	0.25	0.15
8	Cd55	0.37	0.37	23	Ppargc1b	0.24	0.20
9	Mcph1	0.37	0.13	24	Prkdc	0.23	0.09
10	Malt1	0.37	0.06	25	Fzd5	0.22	0.13
11	Nfam1	0.35	0.19	26	Oas3	0.22	0.02
12	Ercc2	0.34	0.15	27	Stk10	0.20	0.06
13	Ddx60	0.33	0.14	28	Cblb	0.20	0.02
14	Itgam	0.33	0.09	29	Tnfrsf1b	0.20	0.06
15	Jak3	0.33	0.18	30	Gpam	0.17	0.03

Data were obtained from four biological repeats and are ranked in order of abundance from highest to lowest. FPKM: Fragments per kilobase of exon model per million mapped reads.

To determine the quality of the total RNAs used for RNA-seq, we determined the RNA Integrity Number of the eight samples. The RINs ranged from 8.7 to 9.5, with an average value of 9.19 ± 0.25, indicating the high quality of our samples.

### Collection of cochlear sensory cell enriched samples

The sensory epithelium collected for RNA-seq analysis included all cell populations in the basilar membrane tissue between the lateral wall and the modiolus of the cochlea (Figure 1A). This tissue contains multiple cell populations, including cells in the organ of Corti partition and cells in the non-organ of Corti partition. Because the organ of Corti partition is susceptible to pathological insults, we sought to determine its immune capacity. We employed micro-dissection to collect the organ of Corti [21]. This tissue contains only sensory cells (inner hair cells and outer hair cells) and their adjacent supporting cells (pillar cells, Deiters cells, inner phalangeal cells and inner border cells) (Figure 1A).

**Figure 2:**
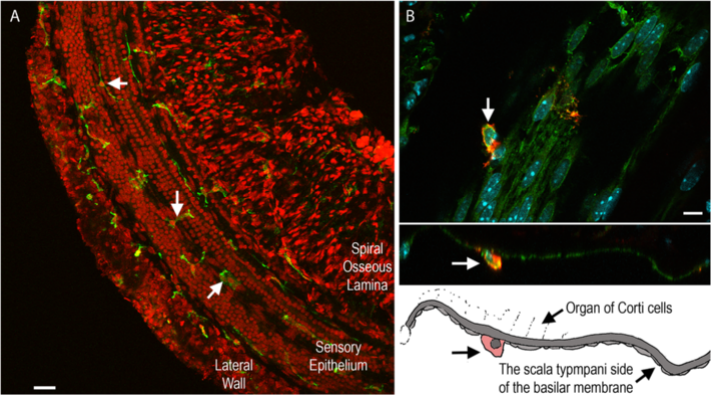
**Typical images of immune cell distribution in the cochlea. (A)** Distribution of immune cells, which were marked by CD45 immunolabeling (green fluorescence marked by arrows) in a whole mount preparation of the cochlea. The tissue was doubly labeled with propidium iodide (red fluorescence), a nuclear dye, to illustrate the tissue structure. Immune cells are present in the lateral wall, the sensory epithelium and the spiral osseous lamina. **(B)** Typical images showing the location of immune cells in the region of the sensory epithelium. Top panel: a surface view of the basilar membrane. The immune cells labeled with CD45 (red fluorescence) reside alongside spindle-shaped mesothelial cells. The arrow points to an immune cell. The middle panel: a side view of the basilar membrane presented in the top panel. The bottom panel: a schematic drawing of the middle panel image. The arrow points to the immune cell illustrated also in the middle panel. Note that this immune cell is located in the scala tympani side of the basilar membrane.

Because the current study focused on the immune capacity of resident cells in the organ of Corti, we sought to ensure that our samples did not contain immune cells. To this end, we used immunocytochemistry to label cells in the sensory epithelium (n = 6 cochleae) expressing CD45. CD45 is a pan-hematopoietic marker for leukocytes that has been previously used for the detection of immune cells in the cochlea [9,10]. This marker was selected because cochlear resident and infiltrated immune cells are all derived from hematopoietic cells [16]. Sporadic CD45 positive cells were found on the scala tympani side of the basilar membrane, as well as on the lateral wall and the osseous spiral lamina (Figures 2A and 2B). No immune cells were detected within the organ of Corti. This observation is consistent with previous observations that the normal organ of Corti lacks immune cells [14]. These data suggest that the organ of Corti tissues collected in this investigation contained only cells from of the organ of Corti.

### Constitutive expression of immune/inflammatory genes in the organ of Corti

To determine whether the genes detected in the cochlear sensory epithelium are also detectable in organ of Corti cells, we used 2 qRT-PCR array plates containing 148 genes related to the immune system and inflammatory processes, including innate and adaptive immunity and inflammatory cytokines and receptors (n = 3 biological repeats). Among these, 45 genes were detected in all organ of Corti samples (30.4% of the genes examined) (Table 3) and 53 genes were not detected in any samples (35.8%, Table 4). The remaining 50 genes were detectable in some, but not all of the samples (34.5%, Table 4). Genes with high expression in the organ of Corti included *Mapk1*, *H2-T23*, *Il6st*, *Irf3*, *Rorc*, *Mif*, *Jak2*, *Stat3*, *Irak1 and Stat1*. These data reveal the robust expression of immune and inflammatory genes in resident cells of the organ of Corti.

**Table 3:** **Expression levels of immune/inflammation-related genes in the organ of Corti (∆Ct values re reference genes)**

**Rank**	**Symbol**	**Mean**	**SD**	**Rank**	**Symbol**	**Mean**	**SD**
1	Mapk1	−2.19	1.08	24	Il10rb	3.90	0.17
2	H2-T23	−1.96	0.82	25	Tlr4	4.05	1.29
3	Il6st	−1.63	0.24	26	Il6ra	4.06	0.82
4	Irf3	−0.06	1.51	27	Nod2	4.45	1.47
5	Rorc	−0.04	0.90	28	Nfkbia	4.48	1.21
6	Mif	0.22	0.22	29	Cx3cl1	4.74	1.21
7	Jak2	0.40	1.04	30	Ifngr1	4.75	0.32
8	Stat3	0.57	0.47	31	Bmp2	4.99	0.79
9	Irak1	0.91	0.49	32	Il15	5.18	0.54
10	Stat1	1.19	1.39	33	Il33	5.58	0.90
11	Vegfa	1.28	0.66	34	Spp1	6.09	2.71
12	Gata3	1.84	0.39	35	Traf6	6.85	0.83
13	Aimp1	2.00	0.24	36	Il1b	6.99	0.95
14	Mapk8	2.15	0.74	37	Lyz2	7.08	2.32
15	Il18	2.48	0.64	38	Tyk2	7.61	0.98
16	Ifnar1	2.50	0.32	39	Tlr3	7.99	0.77
17	Nampt	2.87	0.30	40	Il17a	8.08	2.61
18	Ddx58	2.99	0.69	41	Tnfsf13b	8.49	0.60
19	Nfkb1	3.32	0.31	42	Ticam1	8.68	0.60
20	Ly96	3.32	1.76	43	Irf7	8.78	1.37
21	Tnfrsf11b	3.43	1.58	44	Stat6	10.17	0.56
22	Tlr2	3.47	1.73	45	Nod1	10.57	0.90
23	Il1r1	3.82	1.02				

Data are the means ± SD from three biological repeats and are ranked in the order of abundance from highest to lowest.

**Table 4:** **Genes that were detected in some samples (but not all) or not detected in any samples**

**Detected in some samples**		**Not detected in any samples**	
C3	Il16	Apcs	Cxcl9
C5ar1	Il17b	Ccl1	Cxcr2
Casp1	Il1a	Ccl11	Cxcr3
Ccl17	Il2	Ccl12	Cxcr5
Ccl19	Il23a	Ccl2	Fasl
Ccl5	Il27	Ccl20	Ifng
Ccl7	Il4	Ccl22	Il10
Ccr4	Il5	Ccl24	Il11
Ccr6	Il6	Ccl3	Il13
Cd14	Il7	Ccl4	Il17f
Cd4	Itgam	Ccl6	Il1rn
Cd40lg	Lta	Ccl8	Il21
Cd80	Mbl2	Ccl9	Il2rb
Cd8a	Mpo	Ccr1	Il2rg
Csf1	Mx1	Ccr10	Il3
Csf2	Myd88	Ccr2	Il5ra
Cxcl10	Pf4	Ccr3	Ltb
Cxcl12	Slc11a1	Ccr5	Nlrp3
Cxcl15	Stat4	Ccr8	Osm
Foxp3	Tbx21	Cd40	Rag1
H2-Q10	Tlr5	Cd86	Tlr1
Icam1	Tlr6	Crp	Tlr8
Ifna2	Tlr7	Csf3	Tnf
Ifnb1	Tlr9	Cxcl1	Tnfsf10
Il10ra	Tnfsf13	Cxcl11	Tnfsf11
		Cxcl13	Tnfsf4
		Cxcl5	

Data were obtained from three biological repeats.

We next sought to determine whether genes detected in the organ of Corti were also detectable in the sensory epithelium. Because the organ of Corti is part of the sensory epithelium, we expected that the genes detected in the organ of Corti would also be detected in the sensory epithelium. Furthermore, any genes undetectable in the sensory epithelium should also be undetectable in the organ of Corti. Among the 45 genes detected in the organ of Corti, 44 were also detected in the sensory epithelium. Only one gene (*Nod2*) was not detected in the sensory epithelium. Among 53 genes that were not detectable in any sensory epithelium samples, only the same gene (*Nod2*) was detectable in all samples of the organ of Corti. While the cause of the unexpected expression of *Nod2* is not clear, the consistent results observed for all of the other genes supports the validity of this gene expression analysis.

The sensory epithelium also contains non-organ of Corti cells. The genes in these cells can be identified if they are detectable in the sensory epithelium but not in the organ of Corti. Among the 53 genes that were not detectable in the organ of Corti, 13 were detectable in the sensory epithelium (Table 5). These genes are likely expressed in non-organ of Corti cells.

**Table 5:** **The expression levels of the genes that were detected in the non-organ of Corti portion of the sensory epithelium**

**Symbol**	**FPKM**	
	**Mean**	**SD**
Ccl11	35.24	9.76
Ccl12	30.92	36.56
Ccl6	4.39	1.51
Ccl2	3.87	1.22
Ccl8	2.64	0.87
Ccl9	2.62	1.81
Ccr1	2.51	1.19
Il2rg	2.11	0.84
Ccl24	1.22	1.54
Cd40	0.98	0.52
Cxcr2	0.93	0.43
Cxcl1	0.81	0.80
Tnfsf10	0.67	0.48

Data were obtained from four biological repeats and are ranked in the order of abundance from highest to lowest. FPKM: Fragments per kilobase of exon model per million mapped reads.

### Functional analysis of expressed genes using DAVID

To identify the functional relevance of genes expressed in the organ of Corti, we used two bioinformatics tools, the KEGG-pathway and the Panther-pathway analyses through DAVID [33]. The expressed genes are involved in multiple cellular pathways, including sixteen pathways revealed by the KEGG-pathway analysis and nine pathways revealed by the Panther-pathway analysis. Table 6 lists the genes involved in the top six pathways, as determined by their *P*-values. While there were differences in the pathways identified by the two analyses, both identified the Toll-like receptor signaling pathway as prominent under normal physiological conditions. Based on this finding, we focused subsequent investigations on genes that are related to the Toll-like receptor signaling pathway.

**Table 6:** **The top six pathways and their associated genes detected in the organ of Corti**

**Pathway**	***P-*** **value**	**Gene symbols**
**KEGG-pathway analysis**		
Toll-like receptor signaling pathway	7.7E-19	Traf6, Ifnar1, Irf3, Irf7, Il1b, Irak1, Ly96,Mapk1, Mapk8, Nfkb1, Nfkbia, Spp1, Stat1, Tlr2, Tlr3, Tlr4, Ticam1
Cytokine-cytokine receptor interaction	3.8E-10	Bmp2, Cx3cl1, Ifnar1, Ifngr1, Il1b, Il1r1, Il10rb, Il15, Il17a, Il18, Il6ra, Il6st, Tnfsf13b, Tnfrsf11b, Vegfa
NOD-like receptor signaling pathway	6.8E-9	Traf6, Il1b, Il18, Mapk1, Mapk8, Nfkb1, Nfkbia, Nod1, Nod2
Jak-STAT signaling pathway	5.4E-8	Jak2, Ifnar1, Ifngr1, Il10rb, Il15, Il6ra, Il6st, Stat1, Stat6, Stat3, Tyk2
Cytosolic DNA-sensing pathway	7.3E-8	Ddx58, Irf3, Irf7, Il1b, Il18, Il33, Nfkb1, Nfkbia
RIG-I-like receptor signaling pathway	6.2E-6	Ddx58, Traf6, Irf3, Irf7, Mapk8, Nfkb1, Nfkbia
**Panther-pathway analysis**		
Toll-like receptor signaling pathway	2.2E-14	Traf6, Irf3, Irf7, Il18, Irak1, Ly96, Mapk1, Mapk8, Nfkb1, Nfkbia, Tlr2, Tlr3, Tlr4, Ticam1
Interleukin signaling pathway	2.8E-5	Il10rb, Il15, Il17a, Il18, Il6ra, Il6st, Mapk1, Stat1, Stat6, Stat3
Inflammation mediated by chemokine and cytokine signaling pathway	3.7E-5	Jak2, Cx3cl1, Ifnar1, Ifngr1, Il10rb, Il18, Mapk1, Nfkb1, Nfkbia, Stat1, Stat6, Stat3, Tyk2
Interferon-gamma signaling pathway	3.0E-4	Jak2, Ifngr1, Mapk1, Mapk8, Stat1
JAK/STAT signaling pathway	1.5E-3	Jak2, Stat1, Stat6, Stat3
PDGF signaling pathway	3.8E-2	Jak2, Mapk1, Mapk8, Stat1, Stat6, Stat3

### Protein distribution of immune-related genes in the organ of Corti

To provide further evidence for the expression pattern of immune-related genes in the cells of the organ of Corti, we examined the protein localization of these genes in the organ of Corti using immunohistology. Because immunolabeling is a low-throughput method, we selected four key proteins (IRF7, STAT1, TLR4 and TLR2; n = 3 cochleae for each protein) that are related to the Toll-like receptor signaling pathway. These genes were selected for immunohistological analysis because their transcriptional expression was detected in the organ of Corti (see Table 3).

**Figure 3:**
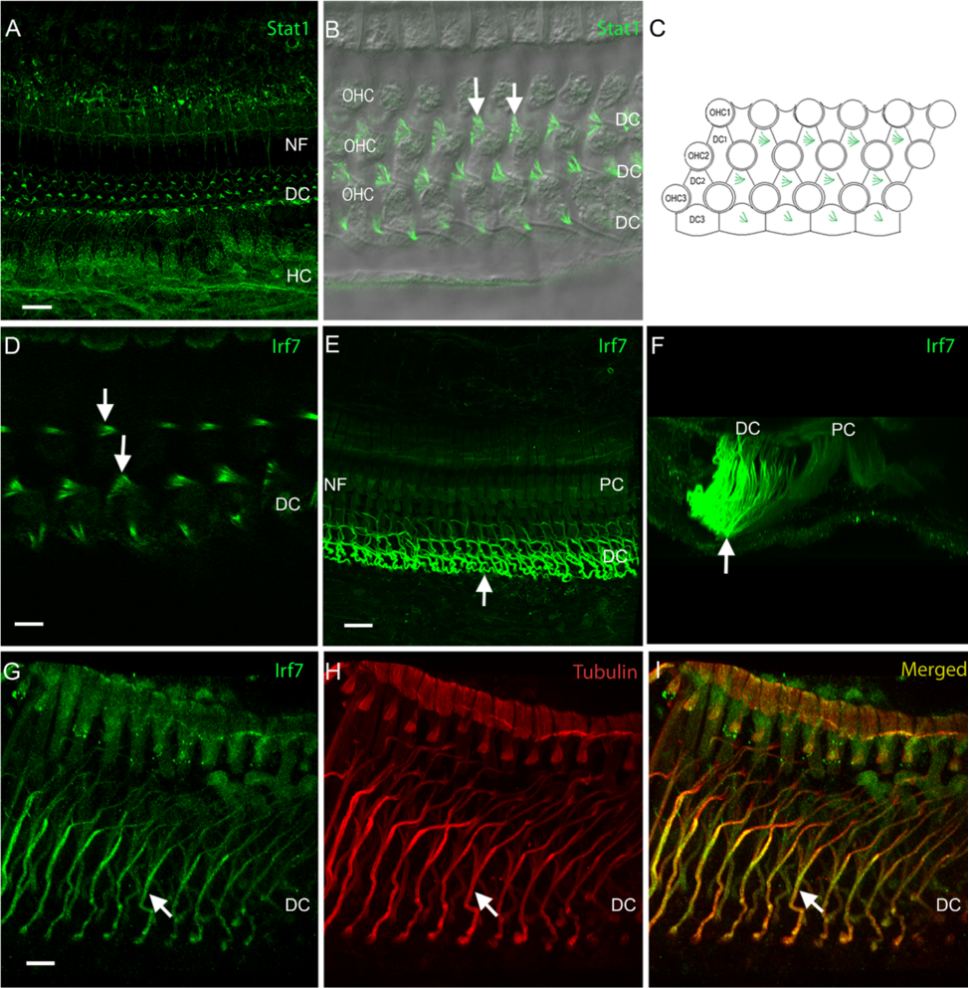
**Immunoreactivity of Stat1 and Irf7 in the organ of Corti. (A)** Stat1 immunoreactivity in the organ of Corti cells. The immunoreactivity is present in the phalangeal process of Deiters cells (also see **(B)** and **(C)** for a high magnification view), Hensen cells, as well as nerve fibers in the inner hair cell region and in the tunnel of Corti. Sensory cells lack immunoreactivity. **(B)** A high magnification view of Stat1 immunolabeling in the phalangeal processes of Deiters cells. The Stat1 immunostaining is superimposed with a differential interference contrast (DIC) view of the tissue to illustrate the contour of the outer hair cells and Deiters cells. Note that Stat1 immunoreactivity is present in the phalangeal processes of Deiters cells (arrows). **(C)** A schematic drawing of the surface view of the organ of Corti mimicking the image **(B)** *.* **(D)** Immunolabeling of Irf7 in the phalangeal process of Deiters cells (arrows). The distribution of Irf7 immunoreactivity in this region is similar to that observed for Stat1. **(E)** Strong immunoreactivity is present in Deiters cell bodies (marked by the arrow). Weak immunoreactivity is present in pillar cells. Sensory cells lack immunolabeling. **(F)** A side view of the image in **(E)** showing the strong Irf7 immunoreactivity in Deiters cells, marked by the arrow. **(G)**, **(H)**, and **(I)** Double-staining of Irf7 and β-tubulin. The Irf7 immunoreactivity and the β-tubulin immunoreactivity are co-localized in the central core of Deiters cells (the arrow). Bar in **(A)** = 20 μm and Bar in **(G)** =15 μm. DC: Deiters cells; HC: Hensen cells; NF: nerve fibers; OHC: outer hair cells; PC: pillar cells.

Confocal microscopy of immunostaining revealed that IRF7 and STAT1 are predominantly found in supporting cells of the organ of Corti. Specifically, STAT1 was detected in Hensen cells, Deiters cells and nerve fibers (Figure 3A). In Deiters cells, STAT1 immunoreactivity was localized to the apical enlargement of the phalangeal process (Figures 3B and 3C). Sensory cells lacked immunostaining.

Strong IRF7 immunoreactivity was clearly visible in Deiters cells. Weak IRF7 immunoreactivity was observed in pillar cells (Figures 3D, E and F). At the top of the phalangeal process, the staining pattern of IRF7 (Figure 3D) was similar to that of STAT1. In the bodies of Deiters cells, IRF7 fluorescence was localized to a stalk-like structure (Figure 3G). This structure resembles the tubulin-rich central core of Deiters cells [36-39]. To confirm this distribution, we double labeled the tissue with an antibody against β-tubulin (n = 3 cochleae). Strong β-tubulin immunoreactivity was found in the central bundle of Deiters cells (Figure 3H), consistent with previously reported observations of β-tubulin labeling [39-41]. Notably, IRF7 immunoreactivity was co-localized with β-tubulin immunoreactivity (Figures 3G, 3H and 3I), suggesting that this immune protein is localized in the central core of Deiters cells. Like STAT1, IRF7 immunoreactivity was not observed in sensory cells.

**Figure 4:**
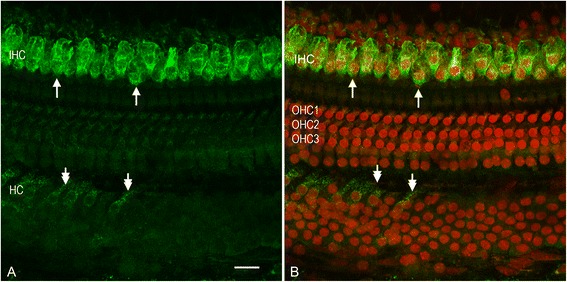
**Immunoreactivity of Tlr4 in the organ of Corti. (A)** A typical image of Tlr4 immunostaining in the organ of Corti. **(B)** Nuclear staining of the same tissue with propidium iodide. Strong Tlr4 immunoreactivity was found in inner hair cells, marked by the top two arrows. Hensen cells display weak immunoreactivity, marked by the bottom two arrows. Outer hair cells and Deiters cells lack immunoreactivity. HC: Hensen cells. OHC1, OHC2 and OHC3: the first, second and third row of outer hair cells. Bar: 20 μm.

Strong TLR4 immunoreactivity was found in inner hair cells (Figures 4A and 4B). Careful inspection of confocal microscopy images showed that the staining was localized to the cell membrane. Hensen cells displayed weaker fluorescence labeling. Outer hair cells lacked immunoreactivity. TLR3 immunoreactivity was not detected in organ of Corti cells under normal physiological conditions (data not shown). Taken together, these observations reveal a cell-specific distribution of Toll-like receptor pathway-related proteins. Interestingly, none of these proteins were detected in outer hair cells, the cell population most vulnerable to common inner ear stresses.

**Figure 5:**
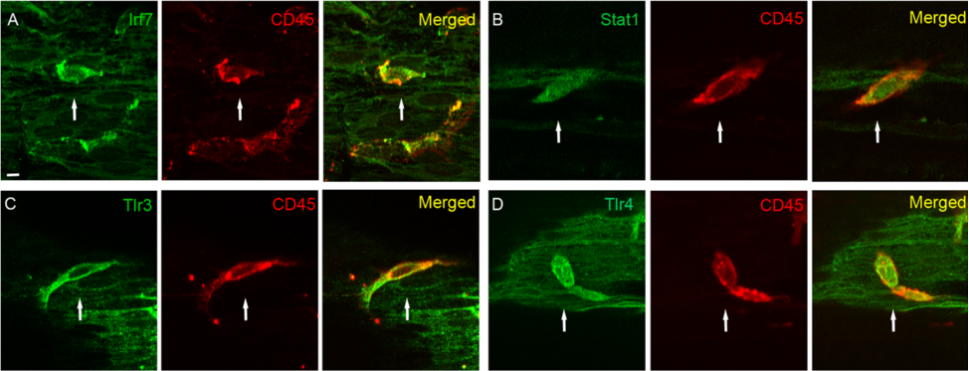
**Immune protein expression in immune cells in the scala tympani side of the basilar membrane. (A)** Double-labeling of Irf7 (green fluorescence) and CD45 (red fluorescence), an immune cell marker. **(B)** Double-labeling of Stat1 (green fluorescence) and CD45. **(C)** Double-labeling of Tlr3 (green fluorescence) and CD45. **(D)** Double-labeling of Tlr4 (green fluorescence) and CD45. The arrow points to an immune cell. Bar =10 μm.

Three methods were used to confirm antibody specificity. First, immune cells in the cochlea acted as a positive control for STAT1, IRF7, TLR3and TLR4 immunolabeling because previous studies have shown that these proteins are expressed in immune cells [42-46]. We found that all four proteins were detectable in immune cells located on the scala tympani side of the basilar membrane (Figure 5). Second, we performed Western blotting to determine the molecular weights of the immune proteins targeted by the antibodies to IRF7, STAT1and TLR3. Using cochlear tissue, we found a 54-kDa band for the IRF7 antibody, which is consistent with its molecular weight. Two bands, of 87 and 170 kDa, were found for STAT1. The 87-kDa band is consistent with the predicted molecular weight of STAT1. The additional band at 170 kDa may be a dimer of the protein [47]. We tested the antibody against TLR3 using brain tissue because cochlear tissues did not yield any clear bands. Three bands were found at 30, 50 and 104 kDa. The 104-kDa band is consistent with the molecular weight of TLR3. The two additional bands may be products of protein cleavage. Finally, we used tissue from TLR4 knockout mice to confirm the specificity of the TLR4 antibody. In Western blot analyses, the knockout mouse did not show Tlr4 protein bands in samples from the spleen, kidney and intestine, whereas the WT mouse tissues did show TLR4 protein bands. Immunolabeling of cochlear sensory epithelium collected from the basal turn of the cochlea in the knockout mouse showed only weak background fluorescence (data not shown).

### Tlr4 knockout does not affect the hearing function of the cochlea

Many immune-related genes have non-immune functions. Given the robust expression of Toll-like receptor signaling genes under normal conditions, we wondered whether this signaling pathway is required for normal hearing. We selected a *Tlr4* knockout model (B6.B10ScN-*Tlr4*^*lps*-*del*^*/JthJ* mice) for evaluation. This strain of mice displays the *Tlr4*^*Lps-del*^ spontaneous mutation, corresponding to a 74,723 bp deletion that completely removes the *Tlr4* coding sequence [48]. Genotyping of the knockout and wild-type mice (C57BL/6 J) revealed a 140 bp product for the *Tlr4* knockout mice and a 390 bp product for the wild-type mice; both are consistent with the expected genotyping results provided by the Jackson Laboratory.

**Figure 6:**
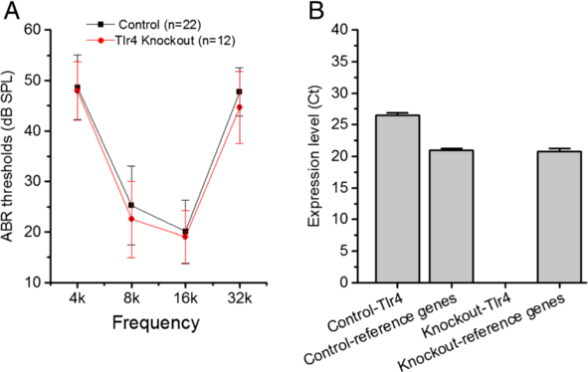
**Maintenance of auditory function in Tlr4 knockout mice. (A)** Comparison of the ABR thresholds between Tlr4 knockout mice and wild-type mice at four tested frequencies. There is no significant difference in the thresholds between the 2 types of mice (2-way ANOVA, *P* >0.05), indicating that disruption of the Tlr4 signaling pathway does not affect auditory function. **(B)** Transcriptional expression levels of Tlr4 in the organ of Corti of Tlr4 knockout and wild-type mice examined by qRT-PCR. The average expression levels of three reference genes (*Hprt1*, *Hsp90ab1*, *Rpl13a*) in the same samples are also present. The expression of Tlr4 was not detected in the Tlr4 knockout mice. This observation confirms that the knockout mice lack Tlr4 expression.

ABR thresholds in *Tlr4* knockout mice (*Tlr4*−/−, n =12 cochleae) were compared with those of wild-type mice (C57BL/6 J, n =22 cochleae) at the age of 4 to 7 weeks. Two-way ANOVA revealed no significant differences in the ABR thresholds between the knockout and the wild-type mice at the four frequencies tested (4, 8, 16 and 32 kHz) (*P* >0.05; Figure 6A). To confirm that *Tlr4*−/−mice lacked *Tlr4* expression in the cochlea, we examined the transcriptional expression of *Tlr4* in cochlear tissue using qRT-PCR (n =3 biological repeats). TLR4 expression was not detected in the knockout mice (Figure 6B). By contrast, the expression levels of three reference genes (*Hprt1*, *Hsp90ab1*, *Rpl13a*) were normal (Student’s *t*-test, *P* >0.05). This suggests that *Tlr4* is not required for normal cochlear hearing.

### Immune genes in the organ of Corti respond to cochlear stress

**Figure 7:**
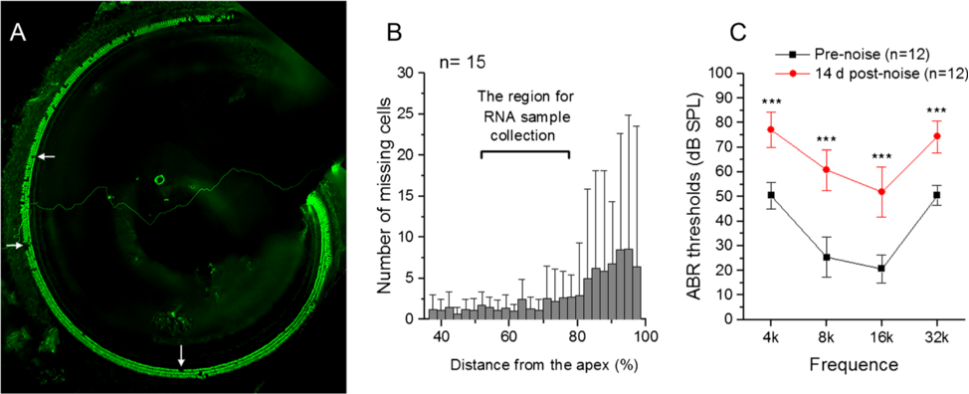
**Structural damage and functional loss of the cochleae examined at 14 days after exposure to an intense noise at 120 dB sound pressure level (SPL) for 1 hour. (A)** Typical pathology of the outer hair cells in the first cochlear turn, from which the organ of Corti tissue was collected for the transcriptional analysis. The tissue was stained for prestin to illustrate outer hair cells. Arrows point to the areas of missing cells. Note that outer hair cell losses are sporadically distributed in the basal turn of the cochlea. **(B)** A cochleogram showing the distribution of hair cell lesions in the basal part of the cochlea (35 to 100% from the apex). The bracket indicates the region from which the organ of Corti tissue was collected for the transcriptional analysis. **(C)** Comparison of ABR thresholds tested before and two weeks after noise exposure. The acoustic overstimulation caused a relatively flat threshold elevation with an average threshold shift ranging from 23.8 to 35.4 dB at the four tested frequencies (2-way ANOVA, F = 403.8, df: 1,88, *P* = 0). n = the number of cochleae used for each condition.

Because we found no detectable impact of *Tlr4* signaling on normal hearing, we focused on determining whether Toll-like receptor pathway genes respond to cochlear stress. We exposed mice to traumatic noise (120 dB sound pressure level (SPL) for 1 hour). To provide a context for interpreting changes in expression, we quantified noise-induced cochlear damage (n = 8 cochleae) and hearing loss (n = 12 cochleae) at 14 days post noise exposure, when noise-induced pathogenesis becomes permanent. Examining cochlear pathology revealed sensory cell loss spread throughout the basal turn of the cochlea, where the samples for gene expression analysis were collected (Figures 7A and 7B). ABR measurements revealed an average threshold elevation of 23.8 to 35.4 dB in the four frequencies tested (4, 8, 16 and 32 kHz) (Figure 7C, 2-way ANOVA, F = 403.8, df 1,88, F = 403.8, *P* = 0).

**Figure 8:**
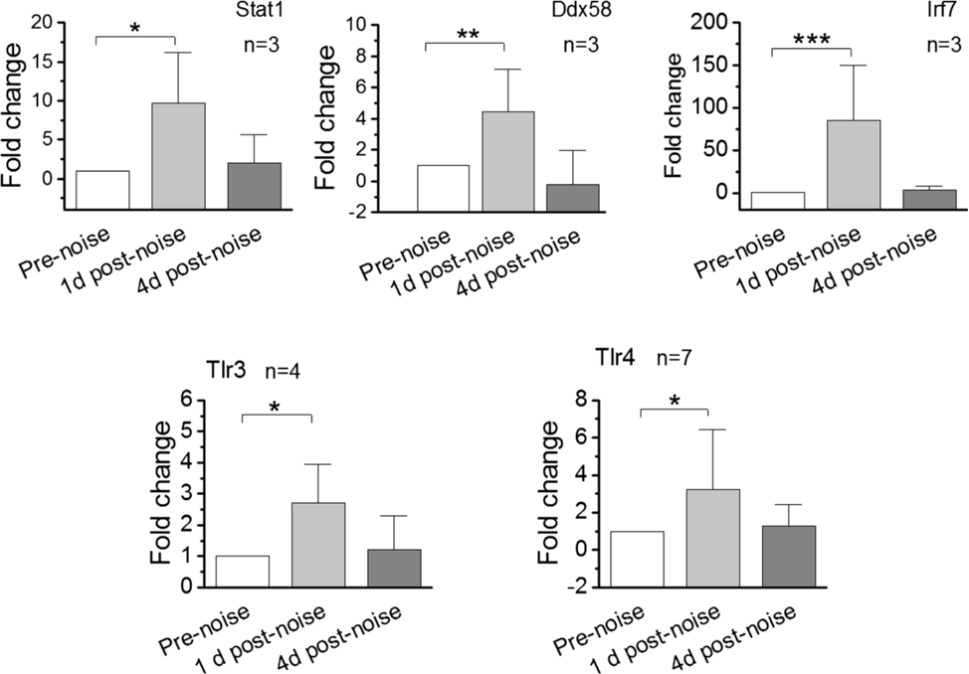
**Expression changes of five genes related to the Toll-like receptor signaling pathway in the organ of Corti after exposure to intense noise at 120 dB sound pressure level (SPL) for 1 hour.** The expression levels were measured 1 day post noise exposure using qRT-PCR. n = the number of biological repeats. Notice that these examined genes display a significant up-regulation at 1 day post noise exposure. *P <0.05, **P <0.01, ***P <0.001.

For the transcriptional analysis, we used a qRT-PCR array to determine the expression levels of 84 immune-related genes, including genes related to the Toll-like receptor signaling pathway, at 1 and 4 days after the noise exposure (n =3 biological repeats). We found that the expression levels of *Irf7*, *Stat1* and *Ddx58* were up-regulated at 1 day post noise exposure and the changes were statistically significant (Figure 8, 1-way ANOVA, F = 38.8(Irf7), 10.7(Stat1), 15.6(Ddx58); df 2,6; *P* = 0.004(Ddx58), <0.001(Irf7), = 0.011(stat1); Tukey test, *P* <0.001 (Irf7), = 0.01(Stat1), = 0.007 (Ddx58). However, this up-regulation disappeared by the fourth day after noise exposure (Tukey test, *P* >0.05), suggesting a time-dependent change in the expression of these genes.

The PCR array analysis also showed a trend towards up-regulation of *Tlr4* and *Tlr3* at 1 day post noise exposure*.* However, this change was not statistically significant (1-way ANOVA, *P* >0.05). We suspect that the lack of significance in this study was due to inefficient power resulting from the small sample size. To verify the trend, we added four new samples collected from four additional mice at 1 day post noise exposure. Individual qRT-PCR analysis revealed a significant up-regulation of *Tlr3* (Figure 8, Student’s *t*-test, *P* <0.05). The change in TLR3 expression was still below the significance level. However, when the PCR array and individual PCR data were combined, the change in the expression level became statistically significant (Figure 8, Student’s *t*-test, *P* <0.05). Together, these analyses implicate the Toll-like receptor signaling pathway in the molecular response of organ of Corti cells to acoustic trauma.

### Changes in Tlr3 and Tlr4 expression are related to sensory cell damage

**Figure 9:**
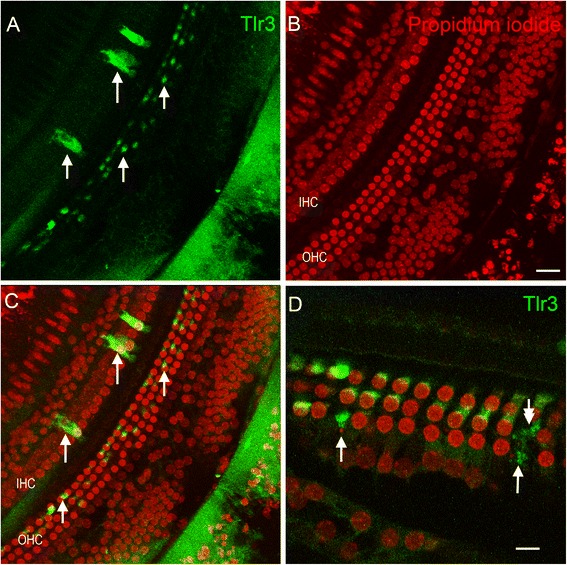
**Typical images of Tlr3 immunoreactivity in the sensory epithelium representing the cochlear section at the distance approximately 70% from the apex.** The tissues were examined 1 day after noise exposure. **(A)** Tlr3 immunoreactivity. **(B)** Propidium iodide staining of the same tissue. **(C)** Superimposed **(A)** and **(B)**. Arrows show the sensory cells with the increased Tlr3 immunoreactivity. **(D)** High magnification view of sensory cells in a section of the basal turn of the cochlea. Arrows indicate an increase in Tlr3 immunoreactivity in outer hair cells with nuclear fragments. The double-arrow indicates an outer hair cell area that has no nuclear staining, suggest degradation of the nucleus. These images show that damaged outer hair cells display an increase in Tlr3 protein immunoreactivity. Bar in **(B)** =25 μm and Bar in **(D)** =10 μm. IHC: inner hair cells. OHC: outer hair cells.

We examined the protein expression of TLR3 and TLR4 in the organ of Corti at 1 day post noise exposure (n = 4 cochleae for each examined protein). This time point was selected because it was the point at which transcriptional changes occurred. Strong TLR3 immunoreactivity, which was not detectable in the normal organ of Corti cells, was found in the cytoplasm of sensory cells (inner and outer hair cells) after noise exposure (Figure 9). However, the staining intensity was not homogenous across cells. In the upper first cochlear turn, where hair cell lesions were present, approximately 42 ± 10% of outer hair cells showed detectable TLR3 immunoreactivity. To define the condition of these TLR3-labeled cells, we doubly stained the tissue with a nuclear dye, propidium iodide. All sensory cells with fragmented nuclei, a sign of apoptosis [1], displayed increased TLR3 immunoreactivity. In areas where the nuclear staining of hair cells was absent, TLR3 immunoreactivity varied. A large portion (approximately 80%) of the area with cells missing their nuclei exhibited increased immunoreactivity. A small portion (approximately 20%) of the area with cells missing their nuclei lacked TLR3 immunoreactivity: an indication of complete degradation of the dead hair cells. These observations suggest that increased TLR3 protein expression is associated with sensory cell damage.

**Figure 10:**
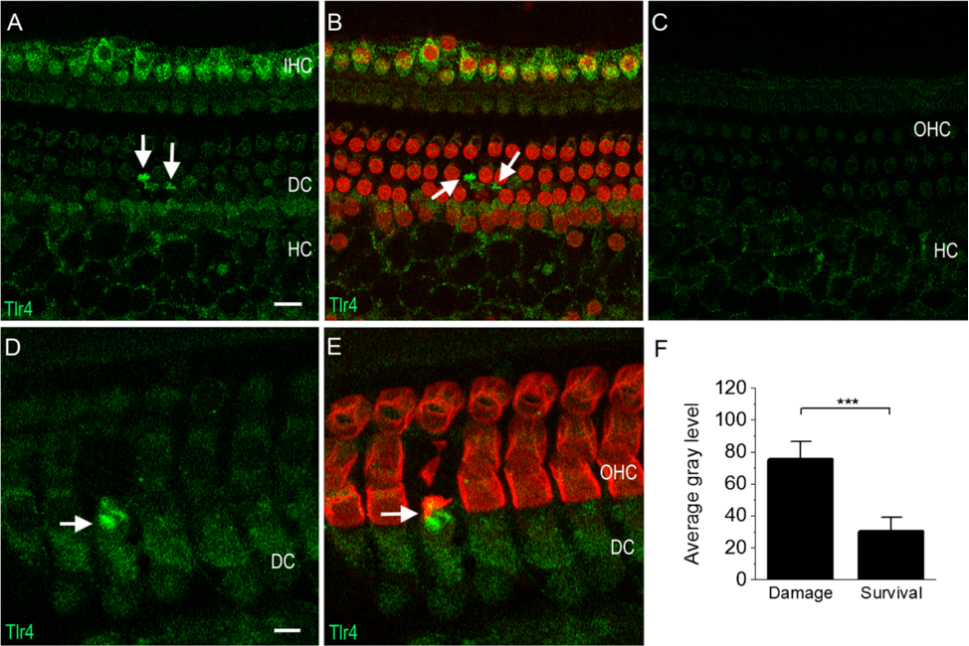
**Typical images of Tlr4 immunoreactivity of the sensory epithelium from cochleae collected 1 day after acoustic trauma. (A)** Tlr4 immunoreactivity is present in inner hair cells and Hensen cells. Arrows indicate increased Tlr4 immunoreactivity in the Deiters cell region. **(B)** Image **(A)** is superimposed with propidium iodide staining to illustrate the nuclear morphology. Notice that Tlr4 fluorescence (marked by the arrows) is located in the Deiters cells adjacent to the areas of missing nuclear staining of outer hair cells, indicating that the increase in Tlr4 in Deiters cells is associated with sensory cell damage. **(C)** A typical image showing that outer hair cells exhibit only weak Tlr4 immunoreactivity. Notice that the image of inner hair cells in this figure is not shown because the inner hair cell image is out of the optical layer of the confocal image. **(D)** and **(E)** A typical example of the increase in Tlr4 immunoreactivity in a Deiters cell beneath a degenerated outer hair cell. The tissue was doubly stained with prestin (red fluorescence), an outer hair cell specific protein, to illustrate the bodies of outer hair cells **(E)**. The arrow in **(D)** and **(E)** indicates an outer hair cell with a malformed cell body, an indication of ongoing degeneration. The Deiters cell beneath this degenerating outer hair cell displays increased Tlr4 immunoreactivity. **(F)** Comparison of the gray level of staining intensity between the Deiters cells beneath surviving outer hair cells and the Deiters cells beneath dying outer hair cells. The average of the fluorescence intensity in the Deiters cells beneath dying outer hair cells is significantly higher than the Deiters cells beneath the surviving outer hair cells (Student’s *t*-test, *P* <0.001). DC: Deiters cells; HC: Hensen cells; IHC: inner hair cells; OHC: outer hair cells.

As in the normal organ of Corti, TLR4 immunoreactivity was observed in inner hair cells and Hensen cells after noise exposure. However, TLR4 immunoreactivity in Deiters cells, which was not observed in the normal organ of Corti, became detectable in some areas after the noise injury (Figure 10A). To determine the condition of cells in the organ of Corti, we doubly stained the tissues with propidium iodide. The immunoreactivity in Deiters cells was located beneath outer hair cells with condensed nuclei or in regions where nuclear staining of the outer hair cells was absent (Figure 10B). To confirm the distribution of TLR4 immunoreactivity in Deiters cells, we doubly stained the tissues with an antibody against prestin (n = 3 cochleae), an outer hair cell specific protein [49]. The sites of TLR4 immunoreactivity and prestin immunoreactivity did not overlap (Figures 10D and 10E), clearly indicating that the staining was specific to Deiters cells. Further quantitative analysis of staining intensity confirmed the difference in TLR4 immunoreactivity between the Deiters cells beneath damaged outer hair cells and the Deiters cells beneath surviving outer hair cells (Figure 10F, Student’s *t*-test, *P* <0.001). This suggests that an increase in TLR4 immunoreactivity in Deiters cells is associated with outer hair cell damage.

## Discussion

The aim of this investigation was to determine the molecular composition of the immune system in the non-immune cells in the organ of Corti and to define the immune responses of those cells to acoustic stimulation. Using RNA-seq and PCR array analyses, we generated global expression profiles of immune/inflammatory genes in both the normal cochlear sensory epithelium and the normal organ of Corti. Bioinformatics analyses revealed an association between the genes expressed and multiple immune signaling pathways, including the Toll-like receptor signaling pathway. Supporting cells were the major anatomic site for the expression of immune genes. Importantly, these genes responded to acoustic overstimulation, and changes in the expression of Toll-like receptor genes were associated with sensory cell damage. Collectively, these results indicate that immune-related genes expressed in the resident cells of the organ of Corti are involved in the cochlear response to acoustic stress.

Molecular changes in the expression of immune/inflammatory genes have been documented in many pathological conditions in the cochlea [4-6,50]. For example, Tra and colleagues have documented that the expression of genes associated with B cell-mediated humoral immune function are altered during age-related cochlear degeneration [51]. These changes have been attributed to several cell populations in the cochlea. Immune cells are a well-known contributor to the cochlear immune system. In response to cochlear injury, circulating immune cells can infiltrate into the cochlea [8,52]. These immune cells release inflammatory mediators and cause inflammatory responses. Fibrocytes, resident cells in the lateral wall of the cochlea, can also participate in the immune response by releasing cytokines and chemokines in response to an immune challenge [53,54]. Here, we provide several lines of evidence that non-immune cells in the organ of Corti are involved in the cochlear immune response to stress.

### Immune related genes are constitutively expressed in the organ of Corti

The sensory epithelium consists of two cell types: organ of Corti cells and the non-organ of Corti cells. Using our recently developed micro-dissection technique, we isolated the organ of Corti cells from the sensory epithelium. We found high levels of constitutive expression of immune genes in both the organ of Corti cells (see Table 3) and the non-organ of Corti cells in the sensory epithelium (see Table 5). The expression of many of these genes has not been previously reported in these tissues.

Several immune/inflammatory genes have been implicated in cochlear pathogenesis [17-20]. Many of these genes were identified in this study. However, some genes found in cochlear tissues during previous investigations were not detected in this study. For example, the expression of IL-6 and IL-1beta in the cochlea has been reported, and their expression is up-regulated in response to acoustic trauma [10,11,55]. These cytokines were not detected in our RNA-seq or qRT-PCR array data. *CD109* is another gene detected in cochlear tissues in a previous study [56] and not detected in this study. These discrepancies may be due to tissue-specific gene expression, although we cannot rule out variations in technique. It is likely that these cochlear genes are also expressed in cells outside of the sensory epithelium that were not included in our analysis: fibrocytes, for example, which are capable of cytokine secretion [53,54].

### Supporting cells are the major site for expression of immune molecules

The organ of Corti contains two types of cell populations, sensory cells and supporting cells. The sensory cells are responsible for converting mechanical energy into neural impulses. These cells are susceptible to stress. Supporting cells provide structural support for the sensory cells and are important for maintenance of the functional and structural integrity of the organ of Corti. Previous studies have documented the expression of immune genes in supporting cells [17-19]. Our immunohistological analyses provide further evidence that supporting cells are the primary site for the expression of immune-genes. Many of the expressed genes were found in Deiters cells, which form direct contacts with outer hair cells. This finding is consistent with our previous observations on the expression pattern of genes related to the complement pathway in the rat cochlea [57]. Deiters cells have been implicated in the recovery process of the organ of Corti in several ways. The phalangeal processes of Deiters cells can expand to seal defects in the reticular lamina where outer hair cells are missing [58]. This repair mechanism is critical for maintaining the ion composition of the endolymph and Corti’s lymph. Deiters cells can also engulf sensory cell debris [59]. Moreover, because of their anatomic location, supporting cells may mediate immune signaling between sensory cells in the organ of Corti and the immune cells located outside of the organ of Corti. This study shows strong expression of *Mif* (macrophage migration inhibitory factor) in cells of the organ of Corti. This gene plays a role in regulating macrophage function in the immune system of non-cochlear tissues [60,61]. In addition to *Mif*, we found expression of TNFα, another immune molecule that is able to recruit immune cells to the cochlea [62]. The constitutive expression of these macrophage-regulatory genes in the organ of Corti suggests a potential role for cells in the organ of Corti to mediate the immune cell response to cochlear stress.

Under normal conditions, outer hair cells do not express the immune proteins we investigated. This suggests that outer hair cells have low immune activity. Lack of the protein expression for these immune-related genes is consistent with the primary function of outer hair cells in cochlear physiology, which is cochlear amplification. Given the strong expression of immune genes in supporting cells, including Deiters cells, we argue that supporting cells are the major players in the immune surveillance of the organ of Corti.

### The Toll-like receptor signaling pathway in cells of the organ of Corti

We performed bioinformatic analyses to determine the functional relevance of the expressed immune genes. Both the KEGG-pathway and the Panther-pathway analyses identified the Toll-like receptor signaling pathway as the leading pathway for immune genes expressed in the organ of Corti. Moreover, Toll-like receptor genes are up-regulated in response to acoustic injury, and these changes are associated with sensory cell damage. The Toll-like receptor signaling pathway plays an important role in innate immunity and can respond to diverse microbial products and endogenous injury-induced molecules [63,64]. The constitutive expression and the rapid up-regulation of these genes after stress suggests a role for the Toll-like receptor signaling pathway in immune surveillance against endogenous sensory cell degeneration after acoustic trauma. Targeting the Toll-like receptor signaling pathway by modulating the expression of *Tlr* genes and their endogenous ligand and signal transduction molecules may be a potential therapy for noise-induced cochlear damage.

This investigation reveals strong TLR3 immunoreactivity in the sensory cells with significant nuclear condensation. This finding raises a question as to why cells display strong immunoreactivity at this late stage of degeneration when, presumably, the protein synthesis machinery has stopped working. It is likely that TLR3 protein synthesis is increased during the early stages of cell damage. The synthesized protein remains in cells even when they enter the later stages of degeneration. This speculation is supported by our observation that increased TLR3 immunoreactivity is present not only in the cells with condensed nuclei but also in cells with normal nuclear morphology. This suggests that increased TLR protein expression is an early event during cell degeneration. Future investigations into the functional role of TLR in sensory cell degeneration and clearance may provide potential therapeutic targets for reducing noise-induced cochlear damage.

Our data support the hypothesis that organ of Corti cells, although lacking in professional immune cells, do have immune capacity. Specifically, we found that immune-related genes are expressed in the resident cells of the organ of Corti and that these genes respond to acoustic trauma. Importantly, we found that immune genes in supporting cells respond to sensory cell damage. These data are consistent with a previous observation that supporting cells engulf dead sensory cells. Together, these data suggest that resident cells in the organ of Corti have immune activity. This activity likely contributes to the clearance of dead cells and to stimulating a cochlear inflammatory response.

Given the potential function of the Toll-like receptor signaling pathway in detecting molecules released from damaged cells, we suggest that activation of this pathway is dependent on the level of sensory cell damage. A high level of noise exposure, as used in this study, can cause sensory cell damage leading to activation of the Toll-like receptor pathway. It is unlikely that low levels of noise exposure, such as those causing temporary hearing loss, can activate this pathway. Additional investigations are required to characterize the noise conditions that activate the Toll-like receptor pathway.

## Conclusions

We have shown the robust expression of immune and inflammatory genes in the organ of Corti. Bioinformatics analyses link these expressed genes to multiple immune-related signaling pathways, including the Toll-like receptor signaling pathway. Importantly, these genes respond to cochlear acoustic stress, and changes in their expression are associated with sensory cell damage. Collectively, these findings show that resident cells in the organ of Corti have immune capacity and are able to participate in the cochlear immune response to acoustic stress.

## Abbreviations

ABR: Auditory brainstem response
FPKM: Fragments per kilobase of exon model per million mapped reads
RNA-seq: RNA-sequencing
SPL: Sound pressure level
